# Research status and trends of diabetic foot related osteomyelitis from 1994 to 2024: A 30-year bibliometric analysis

**DOI:** 10.1097/MD.0000000000046006

**Published:** 2025-11-21

**Authors:** Lei Gao, Tianbo Li, Zixuan Liu, Xinyuan Qin, Jiangning Wang

**Affiliations:** a Orthopedic Department, Capital Medical University Affiliated Beijing Shijitan Hospital, Haidian District, Beijing, China.

**Keywords:** bibliometric analysis, diabetic foot, hotspots, medical information sciences, osteomyelitis

## Abstract

**Background:**

Diabetic foot (DF) is an infectious condition with ulceration and destruction due to neurological and vascular issues. Osteomyelitis affects 10% to 15% of moderate and 50% of severe DF, often requiring surgery and long-term antibiotics. DF related osteomyelitis, resulting from non-healing ulcers, has a high amputation risk and originates from soft tissue infections spreading to bone. However, there are few bibliometric studies on DF and osteomyelitis. This research is to analyze literature on DF and osteomyelitis from 1994 to 2024, exploring research trends and frontiers.

**Methods:**

Literature relating to DF and osteomyelitis from 1994 to 2024 was retrieved from the database of web of science core collection. CiteSpace (v6.3.1) was used to analyze keywords, author information, institution/country information, cited journals and cited references. By constructing a map of co-word, co-citation and cooperation network, the relationship between various literatures was visually displayed, and a visual network map was provided to identify the research hotspots and trends in the field of DF and osteomyelitis.

**Results:**

A total of 2078 valid literatures were retrieved. 140 and 134 studies were published in 2022 and 2023, respectively, showing increased attention to this field. The most productive country and institution were the United States (726) and University of Washington (62), respectively. Aragon-sanchez, Javier (53) was the most productive author. The journal DIABETES CARE (1174) has published the most-cited journal in this field. Lipsky, BA et al [2020] article (143) was the most representative and symbolic reference.

Top 25 keywords with the strongest citation bursts analysis indicates that conservative surgery, wound healing, lower extremity amputations have become research hotspots.

**Conclusions:**

This study explores the research landscape and evolving trends in DF -related osteomyelitis over the last 30 years, offering valuable insights that could assist researchers in identifying important areas of focus.

## 1. Introduction

Diabetic foot (DF) is a significant complication of diabetes, causing various foot problems such as neuropathy, poor circulation, and infections. These issues can lead to ulcers, osteomyelitis, and amputation if untreated.^[1]^ Osteomyelitis, an infection of bone tissue, is a common and serious complication of DF, often due to chronic ulcers or infected wounds.^[2]^ The link between DF and osteomyelitis is complex, involving factors such as peripheral neuropathy, vascular insufficiency, immune dysfunction, and impaired wound healing.^[3]^ Osteomyelitis in DF patients usually requires long-term antibiotic treatment, surgery, or amputation in severe cases.^[4]^

The increasing prevalence of diabetes globally has increased the burden on healthcare systems, with more cases of DF ulcers and osteomyelitis.^[5]^ This has led to more research into understanding the pathophysiology of DF related osteomyelitis, improving diagnostic methods, and developing effective treatments.^[6]^ Advances in both conservative and surgical treatments, including new wound healing strategies and antibiotic therapy, have improved outcomes for patients with these conditions.^[7]^

Bibliometric analysis, using tools like CiteSpace, is crucial for assessing the landscape of DF related osteomyelitis research. It analyzes publication trends, identifies research hotspots, and maps the intellectual structure of the field, offering insights into research topic evolution, scholarly collaboration dynamics, and emerging areas of investigation.^[8–10]^

This study uses bibliometric techniques to systematically analyze the last 30 years research on DF related osteomyelitis. It aims to provide an overview of key contributors, research trends, and the geographical and institutional networks shaping the field. It also highlights areas needing further research, particularly in early diagnosis, infection control, and limb salvage strategies.

## 2. Materials and methods

### 2.1. Data source and search strategy

To identify pertinent studies, a systematic search strategy was employed using the web of science core collection (WoSCC) database.^[11,12]^ The search query was formulated with the following terms: TS = (diabetic foot OR diabetic foot wound OR diabetic foot ulcer OR DFUs) AND TS = (osteomyelitis). The search was limited to English-language articles and review papers published from January 1, 1994, to December 1, 2024, with the data source restricted to the science citation index expanded. To minimize potential biases caused by frequent database updates, the literature retrieval and data extraction were completed on December 27, 2024. A dual-researcher approach was adopted for parallel data extraction and analysis, wherein 2 independent researchers retrieved and cross-verified the literature data, followed by conducting separate visual analyses on the included studies.^[13]^

### 2.2. Data collection and cleaning

The dataset from science citation index expanded includes variables like publication counts, citation metrics, and geographic regions. It was cleaned to remove duplicates and errors. Challenges like multiple reference versions may cause minor discrepancies, but we trust the data’s reliability. A thesaurus improved accuracy before analysis in CiteSpace (6.3.1), VOSviewer (1.6.18) and Excel 2023.^[14]^

### 2.3. Bibliometric analysis

**Figure 1.**
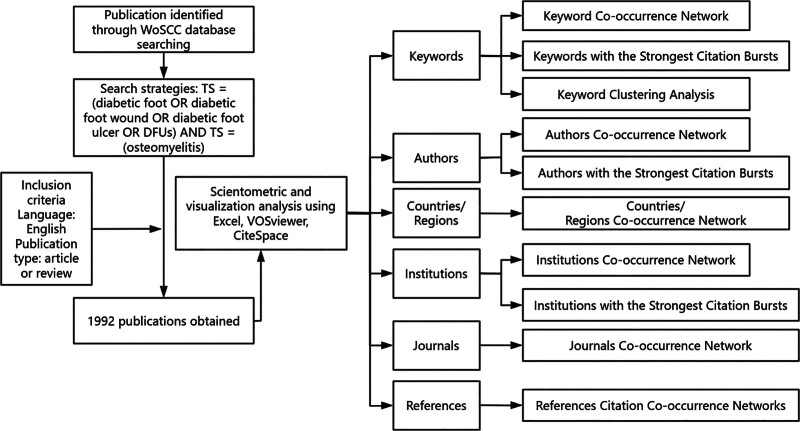
Flowchart of literature search, selection and bibliometric analysis. WoSCC = web of science core collection.

Firstly, at the keyword level, by analyzing keyword co-occurrence relationships, keyword burst trends, and topic clustering, we can identify the research hotspots and emerging trends within the field. CiteSpace generates keyword co-occurrence network maps, revealing the interconnections between research topics as well as the research density and influence of each theme, providing a solid foundation for understanding the core content of the research domain. Secondly, at the author level, by constructing author collaboration network maps and institutional and national affiliation network maps, we can identify the leading scholars in the field and the distribution of their affiliations. This analysis helps to understand which scholars or institutional teams are driving the academic advancements in the field. Finally, at the literature level, we focus on the citation network of significant papers, identifying key seminal works and highly-cited publications that have played a pivotal role in advancing the discipline.^[12,15]^ (Fig. 1).

## 3. Results

### 3.1. An overview of publications on diabetic foot related osteomyelitis

**Figure 2.**
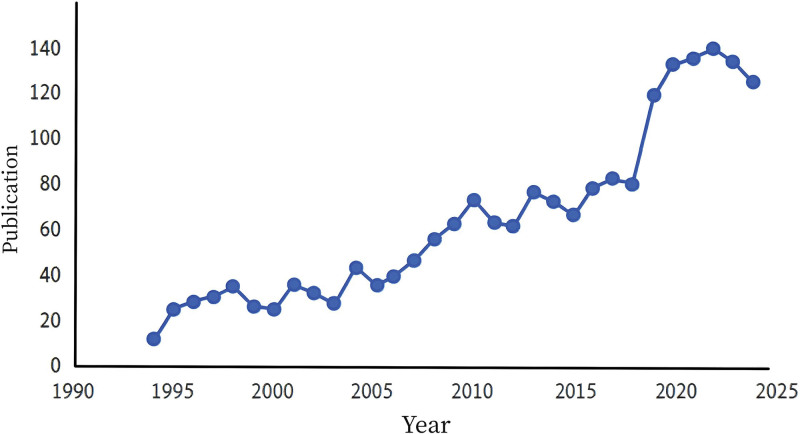
The specific number of annual publications regarding diabetic foot related osteomyelitis from 1994–2024.

Our search strategy yielded 1992 relevant studies published from 1994 to 2024, comprising 1477 articles and 515 reviews (Fig. 2). Since the early 1990s, the volume of relevant literature has gradually increased, with a particularly significant rise in the number of publications after the turn of the 21st century. From 2000 to 2024, the number of publications steadily grew, with a notable peak between 2020 and 2024, where the average annual publication count exceeded 120 articles, reaching 125 in 2024.

### 3.2. The contributions of countries/regions to global publications

**Figure 3.**
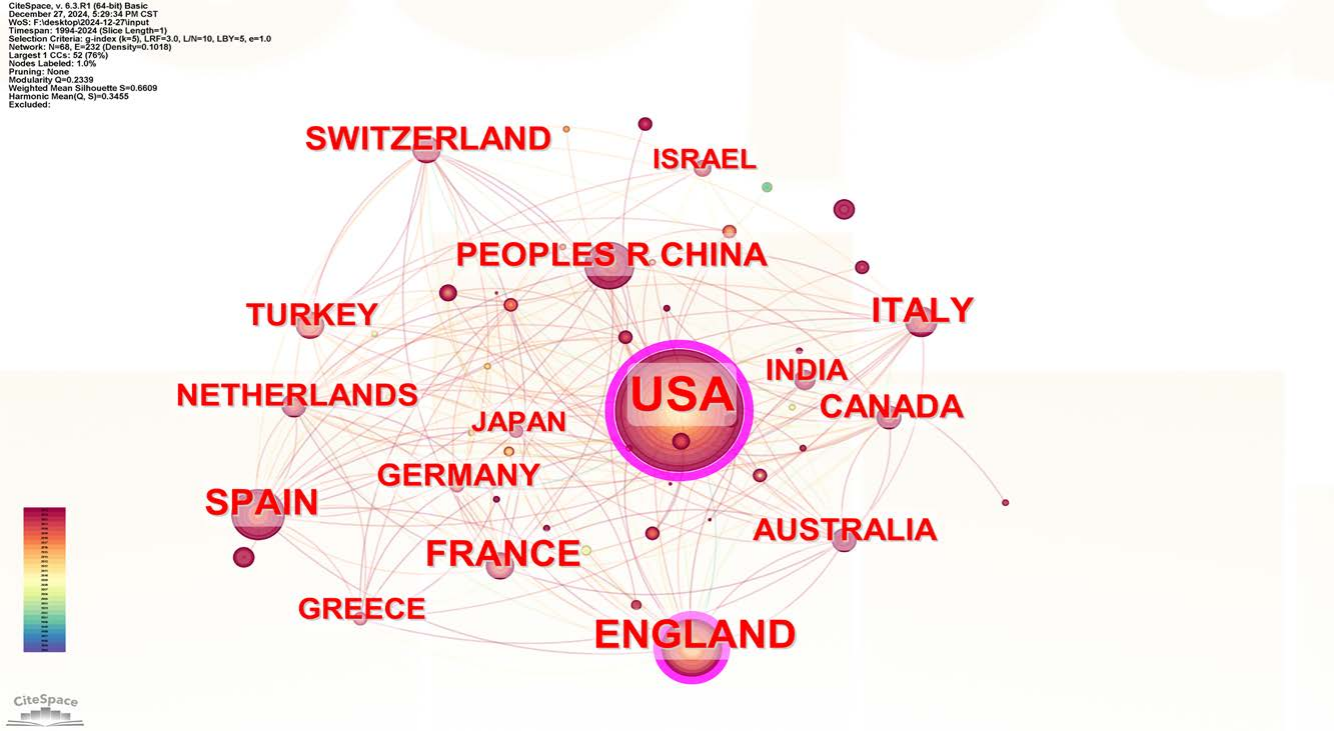
Countries/regions co-occurrence network; In the network map, the nodes symbolize the objects under analysis, with their size corresponding to the frequency of occurrence. The larger the node, the greater the co-occurrence frequency of that node with other surrounding node; the thicker the line, the higher the co-occurrence frequency between the 2 connected nodes. The color and thickness within the node’s inner circle reflect the frequency of occurrence or citation across different time intervals. The edges connecting the nodes and their thickness represent the strength of co-occurrence or co-citation relationships. Nodes with a centrality score >0.1 are highlighted with a purple circle.

The geographic collaboration network, illustrated in Figure 3, comprises 68 nodes and 232 edges. Among these, the United States (Article count = 726) emerged as the leading contributor to DF related osteomyelitis research, followed by England (214), Spain (134), France (132), Italy (97), and China (86). Centrality, a key metric for assessing the significance of nodes within a network, indicates that nodes with higher centrality values play a more pivotal role. The analysis revealed that the United States holds the greatest influence (centrality = 0.46), followed by England (0.23) and Spain (0.1), as detailed in Table 1.

**Table 1:** The top 10 countries/regions contributing to publications in diabetic foot related osteomyelitis research.

Rank	Countries/regions			
	Article count	Degree	Centrality	Name
1	726	38	0.46	USA
2	214	29	0.23	England
3	134	19	0.1	Spain
4	132	18	0.02	France
5	97	19	0.02	Italy
6	86	11	0.01	China
7	83	17	0.02	Switzerland
8	76	21	0.09	Canada
9	68	19	0.08	Australia
10	68	12	0.01	Germany

### 3.3. The contributions of institutions to global publications

**Figure 4.**
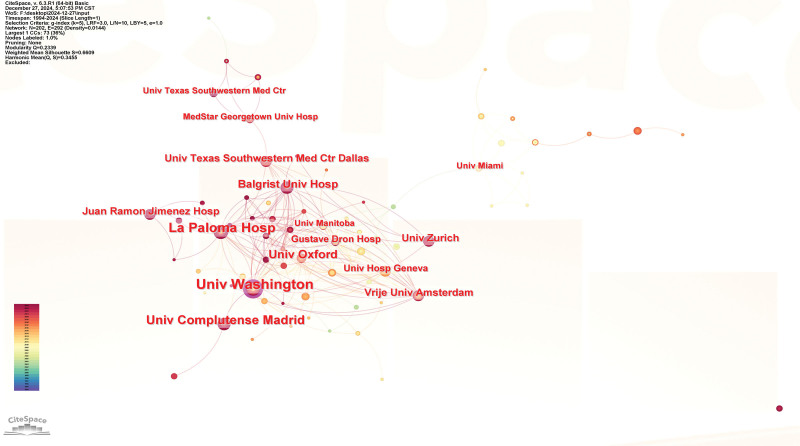
Institutions co-occurrence network; In the network map, the nodes symbolize the objects under analysis, with their size corresponding to the frequency of occurrence. The larger the node, the greater the co-occurrence frequency of that node with other surrounding node; the thicker the line, the higher the co-occurrence frequency between the 2 connected nodes. The color and thickness within the node’s inner circle reflect the frequency of occurrence or citation across different time intervals. The edges connecting the nodes and their thickness represent the strength of co-occurrence or co-citation relationships. Nodes with a centrality score >0.1 are highlighted with a purple circle.

The collaboration network in Figure 4 has 202 nodes and 292 edges. Key contributors to DF-related osteomyelitis research are the University of Washington (Article count = 62), La Paloma Hospital (53), and Complutense University of Madrid (38). Nodes with higher centrality values are more significant in the network. The University of Washington has the highest influence (centrality = 0.12), as shown in Table 2.

**Table 2:** The top 10 institutions contributing to publications in diabetic foot related osteomyelitis research.

Rank	Institutions			
	Article count	Degree	Centrality	Name
1	62	35	0.12	Univ Washington
2	53	27	0.05	La Paloma Hosp
3	38	3	0.01	Univ Complutense Madrid
4	28	14	0.01	Univ Oxford
5	23	13	0.01	Balgrist Univ Hosp
6	20	9	0.03	Univ Texas Southwestern Med Ctr Dallas
7	19	4	0	Univ Zurich
8	16	25	0.02	Vrije Univ Amsterdam
9	16	4	0	Juan Ramon Jimenez Hosp
10	14	8	0	Univ Hosp Geneva

Hosp = hospital, Univ = university.

**Figure 5.**
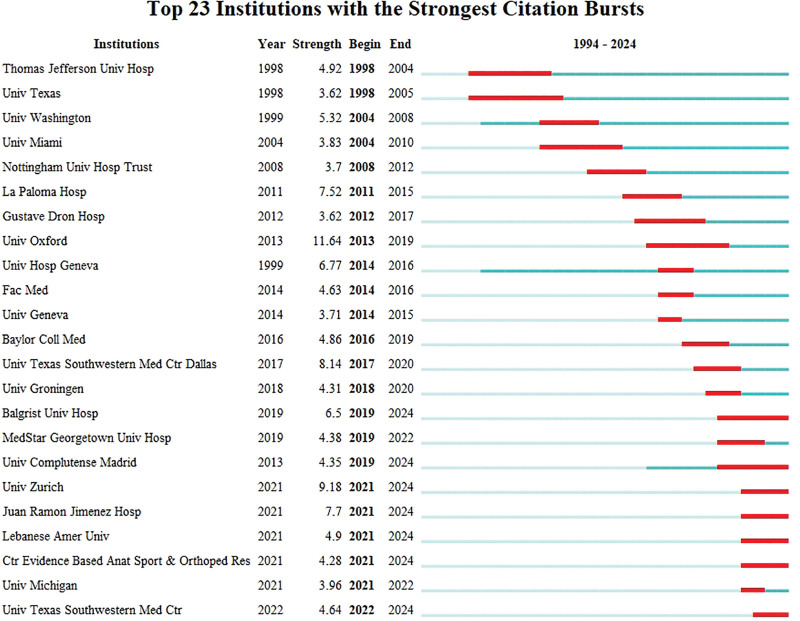
Institutions with the strongest citation bursts analysis. Hosp = hospital, Univ = university.

Institutions with the Strongest Citation Bursts analysis reveals that the University of Washington has emerged as a key player within this academic community. It has become central to the field, likely driving significant research contributions and collaborations. This positions it as a critical institution for shaping future academic and research directions in this domain (Fig. 5).

### 3.4. The contributions of authors to global publications

**Figure 6.**
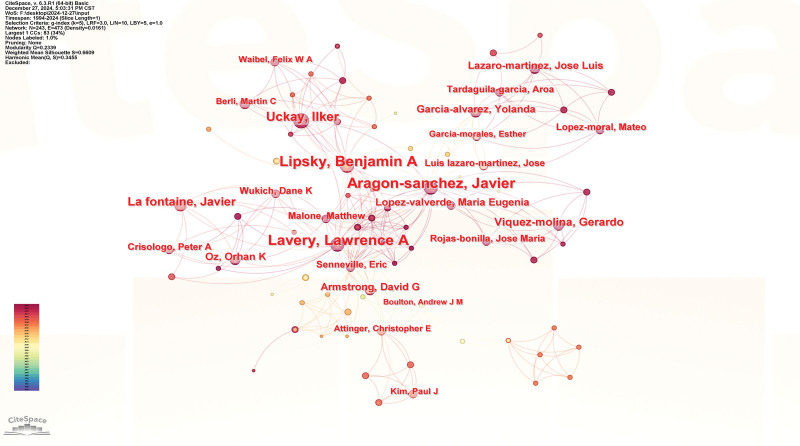
Authors co-occurrence network; In the network map, the nodes symbolize the objects under analysis, with their size corresponding to the frequency of occurrence. The larger the node, the greater the co-occurrence frequency of that node with other surrounding node; the thicker the line, the higher the co-occurrence frequency between the 2 connected nodes. The color and thickness within the node’s inner circle reflect the frequency of occurrence or citation across different time intervals. The edges connecting the nodes and their thickness represent the strength of co-occurrence or co-citation relationships. Nodes with a centrality score >0.1 are highlighted with a purple circle.

The collaboration network depicted in Figure 6 consists of 243 nodes and 473 edges. Leading contributors to research on DF-related osteomyelitis include Aragon-sanchez, Javier (53), Lavery, LA (50), and Lipsky, BA (48). In this network, nodes with higher centrality values hold greater significance. Notably, Aragon-sanchez, Javier and Lavery, LA both exhibit the highest influence, each with a centrality value of 0.1 (Table 3).

**Table 3:** The top 10 authors contributing to publications in diabetic foot related osteomyelitis research.

Rank	Authors			
	Article count	Degree	Centrality	Name
1	53	28	0.1	Aragon-Sanchez, Javier
2	50	29	0.1	Lavery, LA
3	48	23	0.05	Lipsky, BA
4	33	13	0.02	Uckay, Ilker
5	23	7	0	La Fontaine, Javier
6	17	6	0	Viquez-Molina, Gerardo
7	15	8	0	Lazaro-Martinez, Jose Luis
8	15	7	0	Oz, Orhan K
9	14	6	0.01	Armstrong, DG
10	13	10	0.02	Garcia-Alvarez, Yolanda

**Figure 7.**
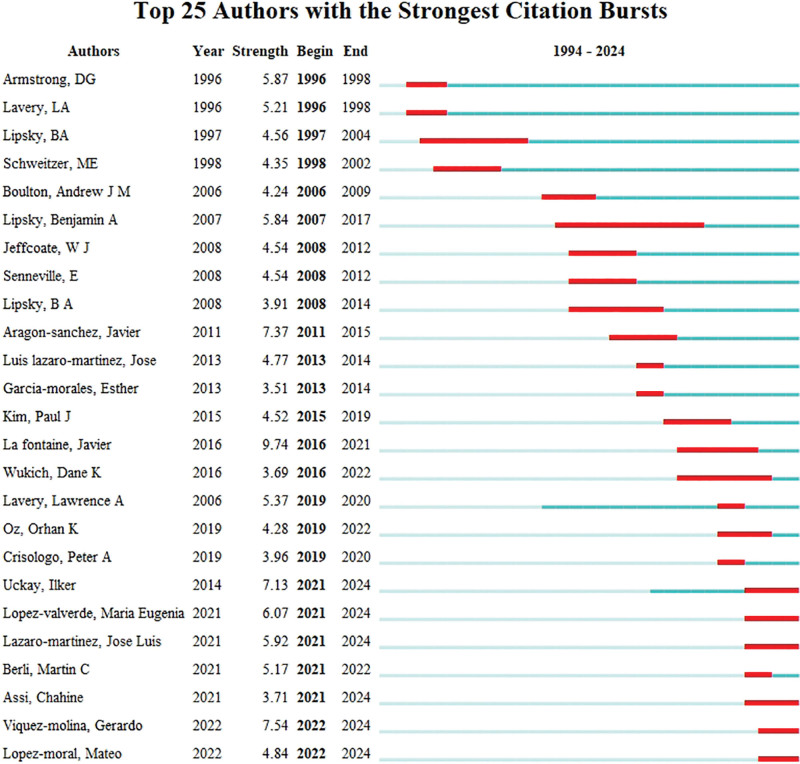
Authors with the strongest citation bursts analysis.

Authors with the Strongest Citation Bursts analysis reveals that Lipsky, BA is one of the most central and influential authors in this field. His contributions have positioned him as a key figure driving research in the area of DF related osteomyelitis (Fig. 7).

### 3.5. Results of co-cited journals network

**Figure 8.**
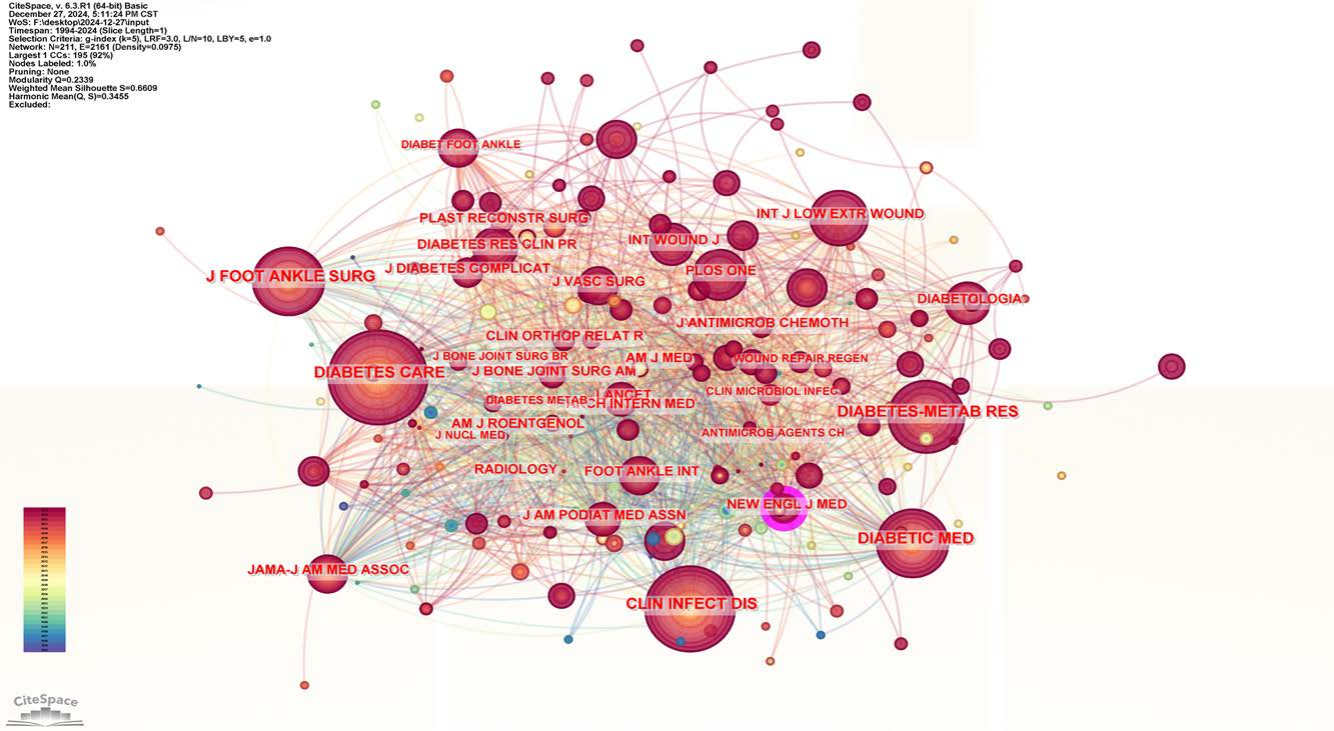
Journals co-cited network; In the network map, the nodes symbolize the objects under analysis, with their size corresponding to the frequency of occurrence. The larger the node, the greater the co-occurrence frequency of that node with other surrounding node; the thicker the line, the higher the co-occurrence frequency between the 2 connected nodes. The color and thickness within the node’s inner circle reflect the frequency of occurrence or citation across different time intervals. The edges connecting the nodes and their thickness represent the strength of co-occurrence or co-citation relationships. Nodes with a centrality score >0.1 are highlighted with a purple circle.

The collaboration network shown in Figure 8 contains 211 nodes and 2161 edges. The frequently co-cited journal on DF-related osteomyelitis include Diabetes Care (1174), Clinical Infectious Diseases (912), and Diabetic Medicine (809). In this network, nodes with higher centrality scores are more significant. Notably, The New England Journal of Medicine demonstrates the greatest influence, with a centrality value of 0.11, as indicated in Table 4.

**Table 4:** The top 10 co-cited journals contributing to publications in diabetic foot related osteomyelitis research.

Rank	Journals			
	Co-cited count	Degree	Centrality	Name
1	1174	64	0.04	Diabetes Care
2	912	58	0.05	Clin Infect Dis
3	809	62	0.05	Diabetic Med
4	764	59	0.06	Diabetes-Metab Res
5	719	54	0.03	J Foot Ankle Surg
6	606	54	0.02	JAMA-J Am Med Assoc
7	595	64	0.08	Foot Ankle Int
8	555	77	0.11	New Engl J Med
9	515	52	0.05	Diabetologia
10	489	49	0.02	Lancet

### 3.6. Analysis of references co-citation network

**Figure 9.**
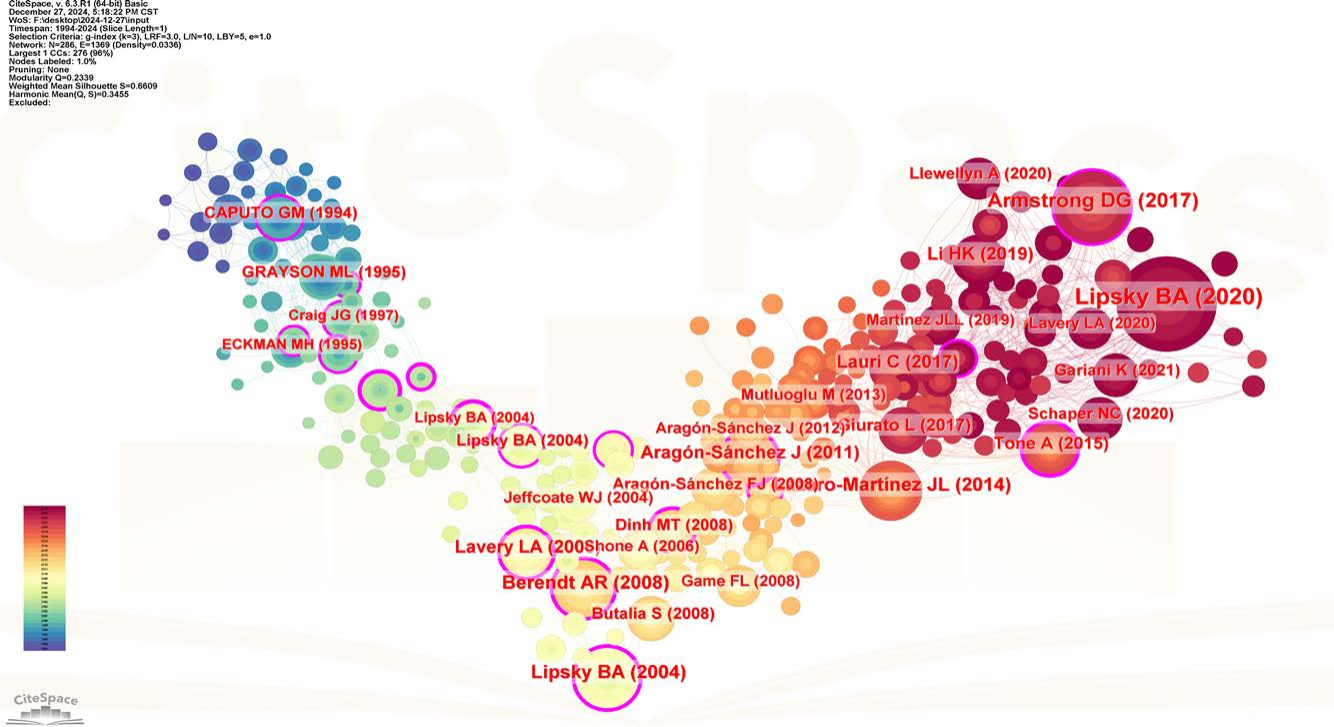
Analysis of references co-citation network; In the network map, the nodes symbolize the objects under analysis, with their size corresponding to the frequency of occurrence. The larger the node, the greater the co-occurrence frequency of that node with other surrounding node; the thicker the line, the higher the co-occurrence frequency between the 2 connected nodes. The color and thickness within the node’s inner circle reflect the frequency of occurrence or citation across different time intervals. The edges connecting the nodes and their thickness represent the strength of co-occurrence or co-citation relationships. Nodes with a centrality score >0.1 are highlighted with a purple circle.

The collaboration network shown in Figure 9 contains 286 nodes and 1369 edges. Table 5 lists the top 10 co-cited references, each with a minimum of 46 co-citations. Of these, 3 had more than 90 co-citations.^[16–18]^ The study “Guidelines on the diagnosis and treatment of foot infection in persons with diabetes (IWGDF 2019 update)” by Lipsky, BA et al, published in “DIABETES-METAB RES” in 2020,^[16]^ had the highest number of cocitations at 143 (Table 5; Fig. 9).

**Table 5:** The top 10 co-cited references contributing to publications in diabetic foot related osteomyelitis research.

Rank	References		
	Co-cited count	Centrality	Co-cited Ref.
1	143	0.07	Lipsky, BA, 2020, Diabetes-Metab Res, V36, P0
2	99	0.3	Lipsky, BA, 2012, Clin Infect Dis, V54, P132
3	94	0.36	Lipsky, BA, 2016, Diabetes-Metab Res, V32, P45
4	83	0.12	Armstrong, DG, 2017, New Engl J Med, V376, P2367
5	58	0.09	Lázaro-Martínez, JL, 2014, Diabetes Care, V37, P789
6	55	0.21	Lipsky, BA, 2004, Clin Infect Dis, V39, P885
7	54	0.27	Berendt, AR, 2008, Diabetes-Metab Res, V24, P145
8	49	0.07	Lavery, LA, 2007, Diabetes Care, V30, P270
9	47	0.07	Li HK, 2019, New Engl J Med, V380, P425
10	46	0.3	Aragón-Sánchez J, 2011, Diabetic Med, V28, P191

### 3.7. Analysis of keywords

**Figure 10.**
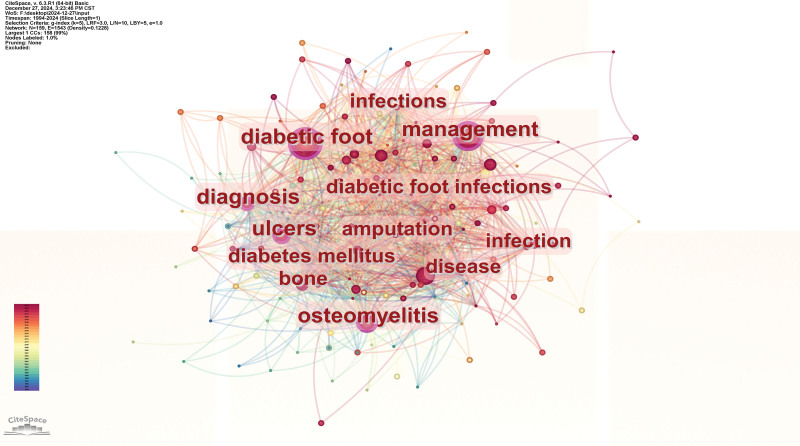
Analysis of keywords co-occurrence network; In the network map, the nodes symbolize the objects under analysis, with their size corresponding to the frequency of occurrence. The larger the node, the greater the co-occurrence frequency of that node with other surrounding node; the thicker the line, the higher the co-occurrence frequency between the 2 connected nodes. The color and thickness within the node’s inner circle reflect the frequency of occurrence or citation across different time intervals. The edges connecting the nodes and their thickness represent the strength of co-occurrence or co-citation relationships. Nodes with a centrality score >0.1 are highlighted with a purple circle.

The collaboration network illustrated in Figure 10 consists of 159 nodes and 1543 edges. The most frequently occurring keywords related to DF and osteomyelitis include DF (462), osteomyelitis (457), and management (350). In this network, nodes with higher centrality values are more prominent. Notably, the keyword ulcers exhibits the highest influence, with a centrality score of 0.19, as shown in Table 6.

**Table 6:** The top 10 keywords contributing to publications in diabetic foot related osteomyelitis research.

Rank	Keywords			
	Article count	Degree	Centrality	Name
1	462	76	0.18	Diabetic foot
2	457	76	0.15	Osteomyelitis
3	350	70	0.14	Management
4	304	68	0.12	Diagnosis
5	296	83	0.19	Ulcers
6	188	58	0.08	Infections
7	156	57	0.07	Amputation
8	146	53	0.09	Bone
9	120	32	0.01	Diabetic foot ulcer
10	119	46	0.07	Diabetic foot infection

**Figure 11.**
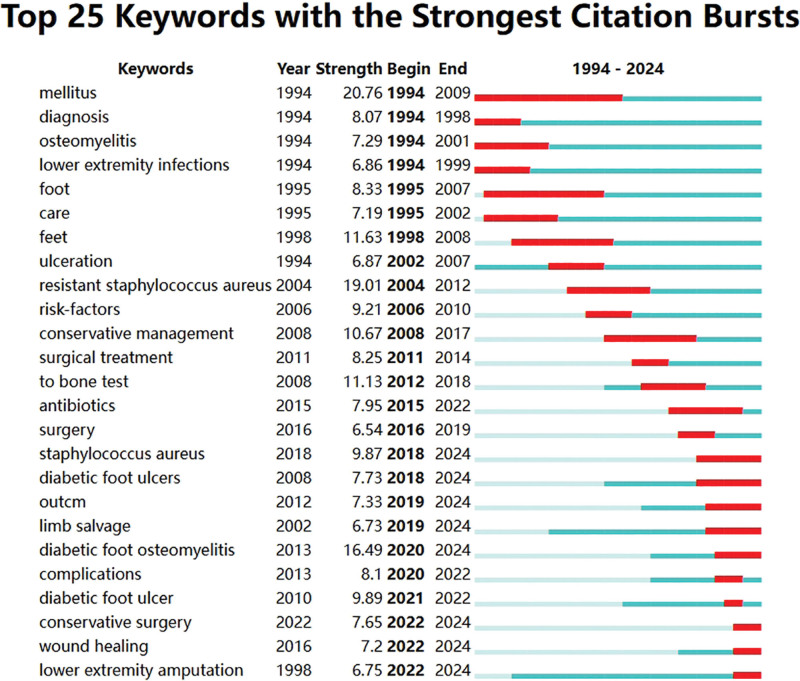
Keywords with the strongest citation bursts analysis.

Keywords with the Strongest Citation Bursts analysis reveals that conservative surgery, wound healing, and lower extremity amputations have emerged as prominent hot topics in the field (Fig. 11).

**Figure 12.**
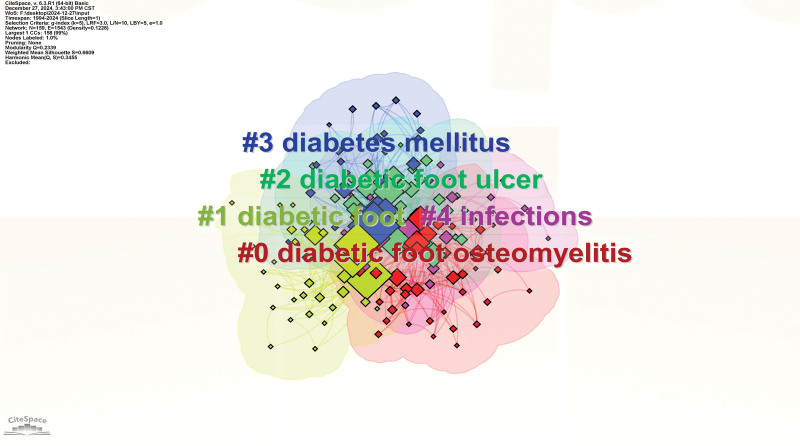
Top 5 keyword clusters of research involving macrophage-related diabetic foot ulcers. The size of nodes represents the co-occurrence frequency, and different colors represent different clustering of keywords.

The extracted keywords were categorized into distinct clusters, with the top 5 based on frequency being “DF osteomyelitis,” “DF,” “DF ulcer,” “diabetes mellitus,” and “infections.” Each of these leading clusters contained a minimum of 20 keywords. Among them, “DF osteomyelitis” had the largest number of keywords (41), followed by “DF” (36) and “DF ulcer” (34). A keyword clustering map was generated based on these 5 most densely populated clusters, where different colors represented distinct keyword categories. Notably, “DF osteomyelitis” exhibited the highest number of nodes and the greatest occurrence frequency (Table 7, Fig. 12).

**Table 7:** The top 5 keyword clusters of research involving diabetic foot related osteomyelitis.

Cluster ID	Size	Silhouette	Mean (yr)	Label (LSI)	Label (LLR)	Label (MI)
0	41	0.536	2011	Diabetic foot; resistant staphylococcus; oral ciprofloxacin; first MTP joint; SPECT imaging diabetic foot osteomyelitis; postoperative antibiotics; first MTP joint; SPECT imaging; peripheral arterial	Diabetic foot osteomyelitis (38.75, 1.0E−4); diabetic foot infections (37.42, 1.0E−4); resistant staphylococcus aureus (28, 1.0E−4); diabetic foot infection (23.52, 1.0E−4); colony stimulating factor (17.12, 1.0E-4)	Fluorodeoxyglucose (FDG) (1.04); diabetic foot infection/ulcer (1.04); classification of diabetic foot infections (1.04); lower extremity wound (1.04); needle bone-biopsy (1.04)
1	36	0.783	1999	Diabetic foot; nuclear medicine; SPECT imaging; diabetes therapy; orthopedics diabetic foot osteomyelitis; diabetic foot infection; diabetic foot ulcers; calcium sulphate; Charcot foot reconstruction	Diabetic foot (34.71, 1.0E−4); magnetic resonance imaging (20.38, 1.0E−4); infection (20.24, 1.0E−4); scintigraphy (19.23, 1.0E−4); unsuspected osteomyelitis (18.54, 1.0E−4)	A venir (1.65); atherosclerosis (1.65); correlation (1.65); classification-system (1.65); anti-granulocyte SPECT/CT (1.65)
2	34	0.582	2009	Diabetic foot; risk factors; total contact cast; glomerular filtration; mellitus diabetic foot ulcer; limb salvage; hyperbaric oxygen therapy; surgical debridement; total contact cast	Diabetic foot ulcer (43.55, 1.0E−4); wound (25.58, 1.0E−4); wound healing (23.8, 1.0E−4); limb salvage (21.17, 1.0E−4); wound care (21.02, 1.0E−4)	Antibiotic susceptibility profiles (1.34); Charcot neuroosteoarthropathy (1.34); foot surgery techniques (1.34); amputation predictors (1.34); foot and ankle infection (1.34)
3	26	0.786	2007	Diabetic foot; osteomyelitis; disease; ulceration; image diabetes mellitus; peripheral vascular disease; foot complications; peritoneal dialysis; neuropathic arthropathy	Diabetes mellitus (44.68, 1.0E−4); mellitus (31.72, 1.0E−4); Charcot foot (24.3, 1.0E−4); Charcot neuroarthropathy (19.55, 1.0E−4); diabetic foot syndrome (16.01, 1.0E−4)	Bone graft (1.24); health care organization (1.24); dynamic study (1.24); circular fixation (1.24); impaired granulocyte adherence (1.24)
4	21	0.669	2010	Diabetic foot; bone infection; oral antibiotics; systemic antibiotics; intravenous antibiotics osteomyelitis; therapy; lower extremity infections; ulceration; limb salvage	Infections (13.85, 0.001); procalcitonin (12.75, 0.001); therapy (10.36, 0.005); septic arthritis (9.91, 0.005); lower extremity infections (8.67, 0.005)	Immunity homeostasis (0.64); bone-targeted therapy (0.64); diabetes cost (0.64); probe to bone (0.64); foot ulcerations (0.64)

CT = computed tomography, LLR = log-likelihood ratio, LSI = latent semantic indexing, MI = mutual information, MTP = metatarsophalangeal joint, SPECT = single photon emission computed tomography.

## 4. Discussion

### 4.1. Annual growth trend analysis

The trend of publications on DF related osteomyelitis indicates a sustained escalation in research interest within the field. Over the past decade, the growth in literature reflects the increasing academic focus on the relationship between DF and osteomyelitis. Particularly in 2022 and 2023, 140 and 134 relevant studies were published, respectively, highlighting the field’s prominence in both clinical and basic research.^[19–21]^ The growing body of literature on DF and its complications, encompassing aspects such as diagnosis, treatment, and prevention, underscores the escalating global health challenge posed by the disease.^[22–24]^ In contrast, the volume of publications before 2000 was relatively low, with only 25 articles published in 2000. As the number of diabetic patients increased and the impact of osteomyelitis on DF patients gained more attention, the volume of related literature began to rise.

### 4.2. Analysis of countries/regions

The majority of highly productive countries/regions are concentrated in Europe and the United States. Through the analysis of the national collaboration network in Figure 10, the following conclusions can be drawn: The United States, England, and Spain are the core countries in the field of DF related osteomyelitis research.^[25]^ They have established close academic collaboration networks with several other countries, including France, Italy, China, Canada, Australia, Germany, and Switzerland.^[26]^ The United States holds the most significant academic influence in this field, with research output and international collaboration capabilities far surpassing those of other countries.^[27]^ The size of the U.S. node is significantly larger than that of the England and China, indicating its leading position in the global academic community. Countries such as China, Italy, and Germany have slightly smaller nodes, but with the enhancement of their research capabilities, their academic status in this field is gradually increasing. Other countries, including France, Canada, Australia, and Switzerland, may have smaller nodes compared to the United States, but their roles in international academic collaboration are indispensable. They have made significant contributions, especially in clinical treatment, diagnostic technologies, and multidisciplinary cooperation.^[28–30]^ Overall, the analysis of the national collaboration network clearly shows that global research on DF related osteomyelitis is developing towards greater internationalization and collaboration. The close cooperation among countries provides a solid foundation for academic progress and innovation in treatment methods in this field.

### 4.3. Analysis of institutions

The institutions co-occurrence network analysis illustrates a dynamic and integrated academic environment for DF and osteomyelitis research, where key institutions from both universities and hospitals collaborate closely (Fig. 4). Among the top ten most productive institutions contributing to this research field, 3 are located in the United States: Univ Washington, La Paloma Hosp, and Univ Texas Southwestern Med Ctr Dallas; 3 are located in Switzerland: Balgrist Univ Hosp, Univ Zurich, and Univ Hosp Geneva; 2 are located in Spain: Univ Complutense Madrid and Juan Ramon Jimenez Hosp; one is located in England: Univ Oxford; and one is located in the Netherlands: Vrije Univ Amsterdam. Institutions like Univ Washington remain dominant, but there are numerous other institutions contributing to and shaping the future of research in this area. The collaborative patterns in the network show a shift toward more interdisciplinary, international, and cross-institutional research partnerships.^[31–33]^

Citation bursts are spikes in the frequency of citations to an institution’s work, signaling academic interest and recognition. Figure 5 lists the top 23 institutions with the strongest citation bursts from 1994 to 2024. Univ Washington experienced a citation burst starting in 1999, peaking around 2005, indicating significant contributions to the field. Univ Texas Southwestern Med Ctr Dallas has maintained a high citation burst since 2018, showing ongoing contributions. Univ Oxford had a high burst starting in 2013, peaking in 2016, suggesting groundbreaking research. La Paloma Hosp had a strong burst starting in 2004, peaking around 2008, indicating early significant contributions. Univ Hosp Geneva has been a strong contributor since 1999. Recent citation bursts from Univ Zurich, Juan Ramon Jimenez Hosp, MedStar Georgetown Univ Hosp, and Baylor Coll Med highlight newer research trends. Ctr Evidence Based Anat Sport & Orthoped Res and Lebanese Amer Univ show recent bursts, signaling rising research contributions. This analysis shows that Univ Washington, Univ Texas Southwestern Med Ctr Dallas, and Univ Oxford have historically dominated research in this field. However, recent citation bursts from Univ Zurich, MedStar Georgetown Univ Hosp, and Univ Texas Southwestern Med Ctr Dallas suggest a shift towards newer contributions and the emergence of rising institutions shaping the academic landscape. Citation bursts reflect the dynamic nature of research in DF and osteomyelitis.^[34–36]^

### 4.4. Analysis of authors

Lipsky, BA is a central figure in the research network, indicating significant collaboration with other researchers, as evidenced by his large node size and strong connections. Aragon-Sanchez, Javier and Lavery, LA are also key figures, with Aragon-Sanchez, Javier’s connections to several authors highlighting his role in advancing DF osteomyelitis research. Lavery, LA’s numerous coauthors reflect his contributions to the field. Smaller sub-networks exist, with authors like Wukich, DK, Boulton Andrew, JM, and Christopher, EA serving as connectors between research areas. The network reveals that a few authors dominate the research landscape, suggesting they are thought leaders in the field. The wide spread of authors across the network signifies international collaboration, showing that research in DF osteomyelitis involves global partnerships. The co-authorship network highlights the central role of authors like Lipsky, BA, Aragon-Sanchez, Javier, and Lavery, LA, emphasizing the interdisciplinary nature of the research and the importance of international cooperation.^[37,38]^

The analysis of the Top 25 Authors with the Strongest Citation Bursts reflects both the established and emerging leaders in the field of DF and osteomyelitis. Long-established researchers like Lavery, LA (1996–1998 and 2007–2017) and Armstrong, DG (1996–1998) continue to lead with sustained academic influence, while newer contributors like Viquez-molina, Gerardo (2022–2024) and Lopez-moral, Mateo (2021–2024) are showing rapidly growing impact, signaling evolving research interests in this domain. This analysis suggests that the field is dynamic, with continuous contributions from experienced researchers and a new generation of scholars contributing significantly to advancing knowledge.

### 4.5. Analysis of journals

Diabetes Care appears as one of the largest nodes in the network, reflecting its strong influence and frequent citations in the research related to DF and osteomyelitis. Journal of Foot and Ankle Surgery (J Foot Ankle Surg) is another prominent journal with significant co-occurrence, indicating its key role in research concerning foot and ankle issues in diabetes. Diabetes Metabolic Research and Reviews is another highly influential publication in the field, especially concerning the metabolic aspects of diabetes and its complications, including DF.^[39,40]^

The network shows a clear connection between diabetes-related journals (e.g., Diabetic Med, Diabetes Care) and surgical/orthopedic journals (e.g., J Foot Ankle Surg, Clin Orthop Relat R), reflecting the intersection of clinical and surgical aspects in DF research. Infectious disease journals like Clin Infect Dis and J Antimicrob Chemother also appear strongly connected to the network, indicating the significant role of infection prevention and management in DF treatment. The collaboration between radiology, wound care journals (Wound Repair Regen, Int J Low Extrem Wound) and diabetes-focused journals emphasizes the multi-disciplinary nature of research involving diagnosis, treatment, and wound healing.

The network reveals interdisciplinary research involving diabetes management, infection control, wound care, and surgical treatment. This highlights the need for integrated approaches in addressing complex conditions like DF and osteomyelitis, with contributions from diverse specialties. Journals such as J Vasc Surg and J Bone Joint Surg Am suggest the important role of vascular surgery and orthopedic surgery in DF care.

The journal co-occurrence network highlights the interconnectedness of various disciplines involved in DF and osteomyelitis research. Key journals like Diabetes Care, The New England Journal of Medicine, and J Foot Ankle Surg play central roles in shaping the research landscape. The collaboration between journals from diabetes care, infectious disease, and surgical treatment fields demonstrates the multidisciplinary nature of this research, aiming to improve patient outcomes through integrated approaches.^[41]^

### 4.6. Analysis of references co-citation network

By analyzing the citation relationships of different articles, a co-citation network is formed, as shown in Figure 12. The citation network of different articles forms a clear “V” shape. The articles located at the center of the “V” are likely key papers connecting different sources, such as Berendt, AR (2008), Lavery, LA (2006), and Lipsky, BA (2004), among others.^[42–44]^ Looking at the left side of the “V,” the relevant articles are from an earlier period, roughly between 1994 and 1997, around the end of the 20th century. This network not only indicates the formation of a substantial citation relationship during the late 20th century but also highlights some important papers from that time, as shown by the red font-marked papers, such as Caputo, GM (1994), Grayson, ML (1995), and Craig, JG (1997) among others.^[45–47]^ The right half of the “V” mainly features some of the more popular articles from recent years, such as Lipsky, BA (2020), Armstrong, DG (2017), and Tone A (2015).^[16,26,48]^ By comparing the blue region on the left and the red region on the right, it is evident that more and more scholars are beginning to engage in research related to this field, and some of the authors in the middle of the “V” are gradually becoming the key figures in the field.

### 4.7. Analysis of keyword and research hotspots

The keyword co-occurrence network is shown in Figure 2. In this network, the larger the node, the higher the co-occurrence frequency with other surrounding keywords. The thicker the lines between nodes, the higher the co-occurrence frequency between the 2 connected nodes. It can be observed that keywords such as management, DF, osteomyelitis, and ulcers appear most frequently, indicating that many articles mention these keywords, and they are closely interconnected. For example, “DF” and “osteomyelitis” are central nodes, closely linked with multiple other keywords, suggesting that these 2 themes are key areas of research. Particularly, the “osteomyelitis” node is large, indicating that osteomyelitis is a very important complication in DF research. “Infections” and “ulcers” are also large nodes, and they are strongly connected to each other, as well as to “DF” and “osteomyelitis,” showing that infections and ulcers are 2 interrelated and crucial issues in the research on DF and osteomyelitis.

Through the analysis of keyword co-occurrence and citation bursts, we can observe the development trends in the field of DF and osteomyelitis research. Below is a detailed analysis of the evolution of this field: Basic Research on Diabetes and Related Diseases (1990s–2000s)^[49,50]^: Keywords like Mellitus (Diabetes) and Diagnosis emerged strongly in the 1990s to early 2000s, highlighting the fundamental role of diabetes as a major factor in DF and osteomyelitis research. Diagnosis became a key focus of research during this period, reflecting the exploration of diagnostic methods and technologies, especially for the early detection of DF and its complications. Keywords like osteomyelitis and ulceration as related complications and clinical manifestations also garnered widespread attention, marking the growing focus on DF complications during this period; Clinical Treatment and Management (2000s–2010s) From the 2000s, the focus of research gradually shifted towards Treatment (e.g., Surgical Treatment, Antibiotics) and Management.^[51,52]^ As clinical treatment methods advanced, scholars began to focus more on surgical treatments, the use of antibiotics, and comprehensive management strategies. The emergence and citation bursts of Surgical Treatment and Antibiotics indicate that clinical intervention became an important component of research in the field. Keywords like Limb Salvage and DF ulcers began to receive increasing attention, indicating the importance of preventing amputations and treating DF ulcers, especially in the long-term care of diabetic patients; Complications and Early Diagnosis (2010s–2020s): In the 2010s, keywords like Complications and Infections saw significant citation bursts. This phase of research began to focus more on DF and its complications (such as osteomyelitis and infections), emphasizing early diagnosis, effective prevention, and comprehensive management. The keywords Diagnosis and Bone also reflect advances in early diagnosis and bone infections (like osteomyelitis), showing that identifying and managing early complications in DF patients had become a research priority^[53,54]^; Personalized Treatment and Innovative Methods (2020s–Present): By the 2020s, research has shifted towards Personalized Treatment (e.g., Conservative Management, Wound Healing) and New Treatment Methods (e.g., DF Osteomyelitis, DF Ulcers). Citation bursts in recent years suggest that treatment for DF has become more personalized and refined, especially in wound healing and conservative surgery. Keywords like Lower Extremity Amputation continue to be a focus of attention, especially given the complications arising from DF and the need for preventive care^[55,56]^; and Current hot topics: Infection and osteomyelitis remain central themes, with growing research on how to effectively address DF infections, especially in the context of increasing antibiotic resistance. Amputation and Limb Salvage remain important topics, reflecting the ongoing need to prevent DF amputations and protect patients’ limbs, with a focus on new surgical treatments and comprehensive management strategies. The close relationship between DF Infections and DF Ulcers is being further defined, emphasizing the complexity and diversity of treatments for DF and osteomyelitis, particularly the interplay between infections and ulcers.^[57,58]^

The research on DF related osteomyelitis has evolved through several stages, from early foundational research on diabetes to the exploration of clinical treatment methods and, more recently, the shift toward personalized treatment and comprehensive management. Researchers are now focusing not only on early diagnosis and prevention of complications but also on new treatment methods to reduce amputation rates and improve patients’ quality of life. Current research trends emphasize comprehensive treatment, early intervention, and personalized care, with particular attention to managing DF infections, ulcers, and their complications.

Through the analysis of keyword clustering based on bibliometric techniques, we can gain insight into the research trends and the development of various subfields within DF related osteomyelitis. Topic 1 is DF osteomyelitis, which primarily includes keywords like resistant staphylococcus, oral ciprofloxacin, first metatarsophalangeal joint, single photon emission computed tomography (SPECT) imaging, DF osteomyelitis, and postoperative antibiotics. DF osteomyelitis is one of the common complications in diabetic patients, often caused by infections. Due to impaired immune function in diabetic patients, it tends to lead to complicated clinical presentations. The mention of resistant staphylococcus in the keywords suggests that osteomyelitis infections may be caused by antibiotic-resistant bacteria, presenting a higher challenge for treatment. The first metatarsophalangeal joint indicates that the disease often occurs in the joint areas of the foot.^[59–62]^ Topic 2 is DF, though the topic name is similar, the included keywords are different, primarily including nuclear medicine, SPECT imaging, diabetes therapy, orthopedics, DF infection, and DF ulcers. These keywords are more focused on the diagnosis and treatment of the disease. For example, advanced technologies such as nuclear medicine and SPECT imaging are used to assess and monitor the pathological changes of DF.^[60]^ Topic 3 is DF ulcer, which includes keywords like risk factors, total contact cast, glomerular filtration, mellitus, limb salvage, and hyperbaric oxygen therapy.^[62–64]^ These keywords indicate that DF ulcers are not only closely related to the pathological mechanisms of diabetes but also involve various factors related to the patient’s overall health status, local treatment methods, and preventive measures. Topic 4 is diabetes mellitus, which includes keywords like osteomyelitis, disease, ulceration, image, peripheral vascular disease, and foot complications. The clustering mainly focuses on the impact of diabetes on foot health, addressing common foot diseases in diabetic patients, such as osteomyelitis, ulceration, peripheral vascular disease, and foot complications.^[65]^ Topic 5 is infections, which includes keywords like bone infection, oral antibiotics, systemic antibiotics, intravenous antibiotics, therapy, lower extremity infections, and ulceration. This reflects the complexity of clinical treatment for bone and lower extremity infections, particularly in selecting antibiotic treatment regimens, managing different types of infections (such as bone infection and lower extremity infections), and considering the multidimensional approaches in different routes of administration. This clustering analysis highlights the interdisciplinary nature of DF and osteomyelitis research, with a focus on infection management, diagnostic techniques, and treatment strategies for DF complications.^[66,67]^

### 4.8. Benefits and limitations

Our study offers several benefits by systematically analyzing DF osteomyelitis publications using a scientific approach, providing a clearer picture of trends for clinicians and researchers. We utilized 4 scientific tools – Excel, VOS viewer, and CiteSpace – for data analysis, ensuring objective and reliable results that boost our study’s credibility.

However, the study has limitations. We only included articles and reviews from WoSCC, not supplementing with other databases or languages. WoSCC is comprehensive, but analyzing multiple databases is difficult. Our study may also have missed recent influential research due to a time-lag effect. We hope to overcome these limitations with future technological advancements in data analysis.

## 5. Conclusion

This study provides an in-depth examination of the research landscape and evolving trends in DF-related osteomyelitis over the past 3 decades. By systematically analyzing a broad array of publications from the last 30 years, the study identifies key shifts in research themes, emerging areas of interest, and evolving methodologies. These insights are crucial for helping researchers and clinicians stay informed about the current state of the field and anticipate future developments. The findings offer a valuable resource for guiding future research directions, identifying gaps in knowledge, and prioritizing areas that hold significant promise for advancing both clinical practices and therapeutic interventions in the treatment of DF-related osteomyelitis. Furthermore, this study lays the groundwork for future investigations by providing a clear overview of the milestones and trends that have shaped the field, enabling the academic community to build upon this foundation with more focused and innovative studies.

## Author contributions

**Data curation:** Tianbo Li, Xinyuan Qin, Jiangning Wang.

**Formal analysis:** Lei Gao, Zixuan Liu, Jiangning Wang.

**Methodology:** Tianbo Li.

**Software:** Lei Gao, Tianbo Li

**Supervision:** Jiangning Wang.

**Writing – original draft:** Lei Gao.

**Writing – review & editing:** Lei Gao.
